# Turbulent jet flow generated downstream of a low temperature dielectric barrier atmospheric pressure plasma device

**DOI:** 10.1038/srep31756

**Published:** 2016-08-26

**Authors:** Richard D. Whalley, James L. Walsh

**Affiliations:** 1 School of Mechanical and Systems Engineering, Newcastle University, NE1 7RU, United Kingdom; 2 Department of Electrical Engineering and Electronics, University of Liverpool, L69 3GJ, United Kingdom

## Abstract

Flowing low temperature atmospheric pressure plasma devices have been used in many technological applications ranging from energy efficient combustion through to wound healing and cancer therapy. The generation of the plasma causes a sudden onset of turbulence in the inhomogeneous axisymmetric jet flow downstream of the plasma plume. The mean turbulent velocity fields are shown to be self-similar and independent of the applied voltage used to generate the plasma. It is proposed that the production of turbulence is related to a combination of the small-amplitude plasma induced body forces and gas heating causing perturbations in the unstable shear layers at the jet exit which grow as they move downstream, creating turbulence.

Low-temperature plasmas are strongly non-equilibrium systems, where the energy of electrons typically exceeds that of the ions and neutrals by several orders of magnitude^1^,^2^; such conditions give rise to striking physical and chemical characteristics. Over the past half-century our growing understanding of these unique systems has underpinned some of the greatest achievements of the modern age, including semiconductor fabrication and energy efficient lighting^3^. The generation of low-temperature plasma under atmospheric pressure conditions presents a new domain in plasma physics and enables the unique physical and chemical properties of the discharge to be accessed beyond the confines of a vacuum chamber.

Of the many different low temperature atmospheric pressure plasma devices currently under investigation, the dielectric barrier plasma plume stands alone due to its inherent ability to produce a plasma which is projected well beyond the confines of the generating electrodes^4^. Such plasmas are typically created by ionizing a flowing noble gas within a capillary which exits into a background gas, creating an inhomogeneous axisymmetric jet flow^4^. The plasma plume, rich in energetic metastable species and electrons, is free to interact with the background gas and in doing so initiates an abundance of dissociation, excitation and ionization reactions, ultimately yielding a rich reactive chemistry which is both accessible and non-thermal. These unique characteristics have been exploited across multiple diverse domains including nanoscale materials modification^5^ where reactive plasma species impinge on a surface to elicit changes in both functionality and morphology, energy efficient combustion where energetic plasma species provide new reaction pathways^6^, and healthcare related applications including wound healing and cancer therapy, where reactive plasma species initiate biological responses in living tissues^7^,^8^.

Underpinning all of these diverse applications is the reactive plasma chemistry. Uncovering a deeper physical and quantitative description of the velocity fields generated downstream of the discharge is the essential first step to improve our understanding of the production mechanisms of reactive chemical species at the plasma-gas interface. In order to address this complex and multifaceted challenge which spans the fields of plasma physics, fluid dynamics and non-equilibrium plasma chemistry, we report on the first quantitative measurements of the velocity fields generated downstream of a low temperature atmospheric pressure plasma plume.

## Results and Discussion

The turbulent flow field of variable-density jets is characterized by the Froude number (*Fr*), which is the ratio of momentum flux to buoyancy flux. The Froude number is defined as 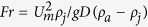, where *ρ*_*j*_ is the density of the injected fluid, *ρ*_*a*_ is the density of the ambient fluid and *g* is the acceleration due to gravity. The vertical flow field is divided into three sections^9^, which are functions of downstream distance (*x*). First there is a region of pure jet behaviour which is dominated by momentum flux. This region begins at the jet exit (*x*/*D* = 0, where *D* is the inner diameter of the capillary) and extends as far as the dimensionless abscissa, X_I_ = (x_I_/D)Fr^−0.5^(ρ_j_/ρ_a_) ^−0.25^ ≈ 0.5. The pure jet transitions into a buoyant jet in an intermediary region which extends to ~10*X*_*I*_. Beyond the intermediary region, the flow is dominated by buoyancy. The data presented here predominantly resides in the pure jet region where buoyancy is negligible: see Table 1 which details the downstream extent of the pure jet region, *x*_*I*_/*D*.

**Figure 1:**
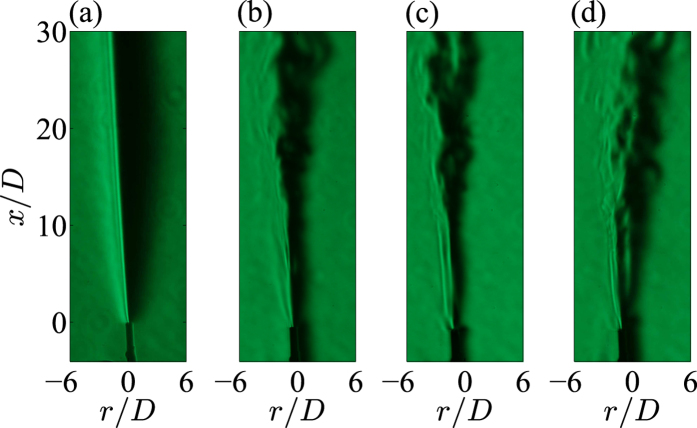
Schlieren photography of (**a**) helium jet flow at *Re* = 135 and (**b–d**) helium jet flow at *Re* = 135 with plasma ignited at (**b**) 10 kV, (**c**) 13 kV and (**d**) 16 kV.

Figure 1 shows Schlieren photography of the axisymmetric helium jet flow at a Reynolds number of *Re* = *U*_*m*_*D*/*ν*_*j*_ = 135 (where *U*_*m*_ is the jet exit velocity and *ν*_*j*_ is the kinematic viscosity of helium). The jet flow begins at *x*/*D* = 0 and *r* is radial distance. In the absence of plasma (Fig. 1a) the helium jet flow remains laminar. This is entirely consistent with observations at *Re* = 260, and is broadly consistent with the flow fields at *Re* = 400, which begin to transition into a turbulent regime at *x*/*D* ≈ 25. These observations are in contrast to the instantaneous flow fields of the helium jet flow with plasma ignited, Fig. 1(b–d), where density fluctuations generated by the application of the plasma indicate a transition-to-turbulence from *x*/*D* ≈ 10. The developing turbulent flow fields observed in Fig. 1(b–d) are entirely consistent with all others in the literature^10^,^11^,^12^,^13^; the turbulent flow fields generated downstream of atmospheric plasma plumes have been qualitatively observed over a wide range of Reynolds number (135–840), applied voltage (8–30 kV peak-peak) and frequencies (125–50,000 Hz; *St*_*θ*_ = *fθ*/*U*_*m*_ = 0.000012–0.035, where *θ*(~0.01*D*) is the momentum thickness of the shear layers at the jet exit).

**Figure 2:**
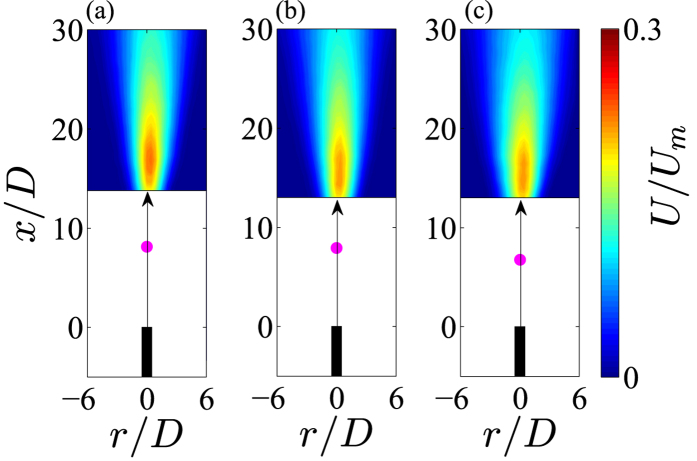
Ensemble-averaged streamwise velocity of helium jet flow at *Re* = 260 with a plasma ignited at (**a**) 12 kV, (**b**) 14 kV and (**c**) 16 kV. Pink dot marks the end of the plasma plume.

Figure 2 shows ensemble-averaged streamwise velocity of helium jet flow at *Re* = 260 with a plasma ignited at 12–16 kV peak-peak. We observe that increasing the applied voltage does not increase the magnitude of the ensemble-averaged streamwise velocity within the turbulent flow region downstream of the discharge. This observation is entirely consistent with data at *Re* = 135 and 400. Note that throughout Fig. 2, the location where the visible plasma ends is marked with a pink dot, and data in the laminar flow regime is omitted due to lack of PIV seeding.

The ensemble-averaged electro-hydrodynamic force generated by guided streamers is ~5 × 10^3^ Nm^−3^ 14. Assuming, conservatively, that each guided streamer has a sheath width of ~250 μm^14^, the increase in velocity due to the electro-hydrodynamic body force is ~0.003−0.025*U*_*m*_ over *Re* = 135–400. Further, the plasma increases the mean temperature of helium at the jet exit by ~10–30 degrees Celsius at 10–16 kV over *Re* = 135–400. Such a change in temperature causes an increase in jet exit velocity of ~0.03–0.09*U*_*m*_. Thus, gas heating and electro-hydrodynamic body forces do not significantly alter the mean velocity fields.

**Figure 3:**
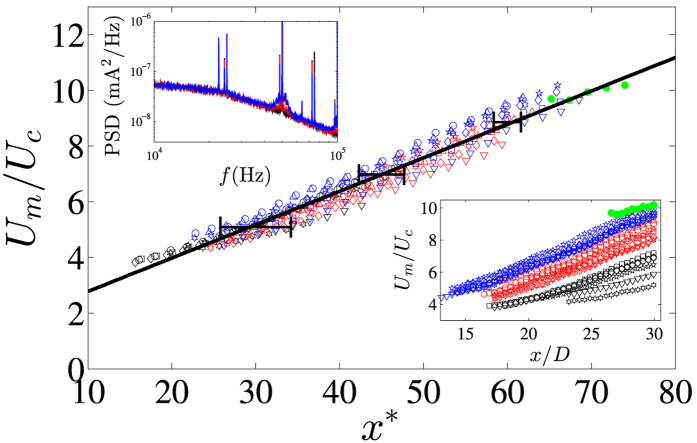
Streamwise decay of the ensemble-averaged centreline velocity plotted against x^*^. Inset (bottom right) plotted against *x/D*; 10 kV (Δ), 11 kV (∇), 12 kV (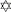), » 13 kV (☆), 14 kV (◻), 15 kV (◇), 16 kV (○); *Re* = 135 (black symbols), *Re* = 260 (red symbols), *Re* = 400 (red symbols). The green closed circles are helium jet flow without plasma at *Re* = 400 and *Fr* = 38,800. Inset (top left) shows power spectral density of current data at *Re* = 260; 12 kV (black line), 14 kV (red line), 16 kV (blue line). The data in the central part of the figure are down-sampled by a factor of two to ease viewing. The error bars show the uncertainty in obtaining the virtual origin.

It is well known that the shear layers at the exit of an axisymmetric jet are highly unstable^15^,^16^. Small finite perturbations (~0.1*U*_*m*_) over a wide frequency range (*St*_*θ*_ < 0.04) can grow exponentially in the unstable shear layers to transition jet flows into a turbulent regime from *Re* > 50^17^,^18^,^19^,^20^. Indeed, linear stability theory^19^ and many experimental investigations^21^ of homogeneous axisymmetric jet flow predict a dominant excitation frequency (*f*_0_) at a Strouhal number of *St*_*θ*_ ≈ 0.017. In this investigation at a *Re* = 260, a *St*_*θ*_ ≈ 0.017 gives a dominant excitation frequency of *f*_0_ ~ 51 kHz (see Table 1), which is close to the driving frequency of the sinusoidal waveform used to generate the plasma. More recently global instability modes in low density axisymmetric jets^22^ have been found to scale as *fD*^2^/*v*_*j*_ ~ *Re*(*D*/*θ*_0_)^1/2^(1 + (*ρ*_*j*_/*ρ*_*a*_)^1/2^), entirely consistent with dominant frequencies on the order of 10’s kHz for the helium jet flows discussed here, albeit with them located in the lower limit of this scaling law. Indeed, energy spectra of the discharge current show a dominant peak at the driving frequency of *f* ≈ 50 kHz, accompanied by multiple high energy harmonics: see the (upper left) inset of Fig. 3. Thus, we propose that the sudden onset of turbulence observed within the flow fields (Fig. 2(b–d)) is generated by a combination of small-amplitude electro-hydrodynamic body forces and gas heating causing perturbations in the unstable shear layers at the jet exit, which become amplified as they travel downstream causing velocity fluctuations, Reynolds shear stresses, and thus the production of turbulence. Moreover, at higher applied voltages, the energy injected into the developing shear layers at the jet exit is increased, see for example the increase in energy around *f* ≈ 50 kHz in the (upper left) inset in Fig. 3, which causes the onset of turbulence closer to the jet exit; an observation well known within the literature^10^,^11^,^12^,^13^, and one which is *not* accompanied by any significant increase in jet velocity, see Fig. 2. The reduction in plasma plume length for higher applied voltages (see for example the locations of the pink dots in Fig. 2) is also due to the production of turbulence, mixing air into the helium jet flow and quenching the discharge.

Finally, we characterize the turbulent velocity fields generated by the atmospheric pressure plasma plumes. Figure 3 shows excellent collapse of the decay of the ensemble-averaged centreline velocity (*U*_*c*_) when plotted with reduced streamwise coordinate, x^*^ = (*x* − *x*_0_)/*d*_*e*_(*x*)^23^. The decay of the ensemble-averaged centerline velocity with increasing distance downstream from the jet exit does not depend on applied voltage. The linear fit to the data in Fig. 3 has gradient, *K* = 0.120 ± 0.017 (to one standard deviation), which is in excellent agreement with inhomogeneous jet flow data in the literature^9^,^23^,^24^. The error bars show the uncertainty in obtaining the virtual origin (*x*_0_), which is ultimately due to the spatial resolution of the PIV setup. For comparative purposes, data from a helium jet flow at *Re* = 400 with no discharge is also included (green closed circles), and shows the same rate of decay of the ensemble-averaged centerline velocity. For reference, the unscaled mean centreline velocity is shown on the (lower right) inset in Fig. 3. The jet-half width scales as *r*_1/2_ ~ *x*, a linear fit to the data provides the virtual origin. Noteworthy is the jet half-angle, tan(*β*) ≈ 0.14, which is approximately double the typical angle found in the literature^23^,^25^, most likely due to a low Reynolds number effect^26^. The local effective diameter, *d*_*e*_(*x*) = *D*(*ρ*_*j*_/*ρ*_*e*_)^1/2^, accounts for the density gradient along the centreline of the developing inhomogeneous jet flow. Djeridane *et al.*^27^ showed that the axial evolution of local density within the pure jet region of helium and carbon dioxide axisymmetric jets scales as *ρ*_*e*_ ~ *K*_*c*_(*ρ*_*j*_/*ρ*_*a*_)^−0.6^(*x* − *x*_*c*_)/*D*, albeit over *Re* ~ 10^3^–10^4^. Here *K*_*c*_ = 0.133^9^,^27^ is a constant, and *x*_*c*_ is the length of the potential core of the jet, which is assumed to be negligibly small^28^.

**Figure 4:**
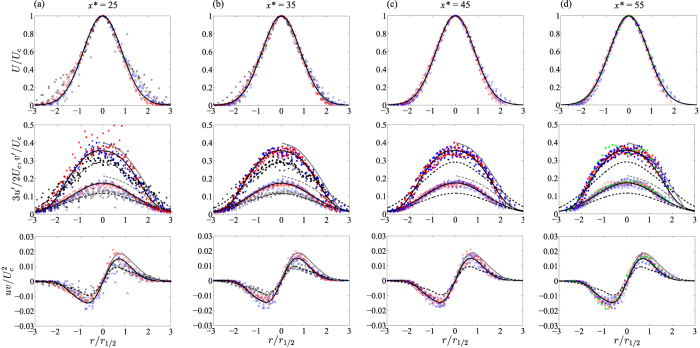
Self-similar radial profiles of ensemble-averaged normalised turbulence statistics at (**a**) x^*^ = 25, (**b**) x* = 35, (**c**) x* = 45 and (**d**) x* = 55 showing (top) mean streamwise velocity with a Gaussian fit of the form: exp(−*r*/*r*_1/2_)^2^ ln(2), (middle) streamwise turbulence intensity (closed symbols) with least-squares spline fits to guide the eye, and radial turbulence intensity (open symbols) with Gaussian fits of the form: 0.174 exp(−0.4*r*/*r*_1/2_)^2^ ln(2) at *Re* = 135 (black symbols), and 0.117 exp(−0.33*r*/*r*_1/2_)^2^ ln(2) at *Re* = 260 (red symbols) and at *Re* = 400 (blue symbols), and (bottom) Reynolds shear stress with least-squares spline fits to guide the eye; 10 kV (Δ), 11 kV (∇), 12 kV (), 13 kV (☆), 14 kV (◻), 15 kV (◇), 16 kV (○); helium round jet turbulence data (*Re* = 7000, *Fr* = 643) from Amielh *et al.*^24^ (grey solid and dashed lines). For further comparison, the green closed circles in (**d**) are helium jet flow without plasma at x^*^ = 70: *Re* = 400 and *Fr* = 38,800.

Figure 4 shows the self-similar turbulence structure of the developing inhomogeneous axisymmetric jet flow at *Re* = 135 (black symbols), *Re* = 260 (red symbols) and *Re* = 400 (blue symbols). With increasing distance from the jet exit, the radial profiles of the ensemble-averaged streamwise velocity (top) collapse to a single normalised exponential curve of the form: *U*/*U*_*c*_ = exp(−*r*/*r*_1/2_)^2^ ln(2), which is entirely consistent with all other turbulent jet data in the literature^9^,^16^,^23^,^24^. The streamwise (*u*′; middle) and radial (*v*′; middle) velocity fluctuations, which represent local turbulence intensities, and the Reynolds shear stresses (*uv*; bottom), which indicates turbulence production, collapse when x^*^ > 35. The radial velocity fluctuations at *Re* = 260 and 400 follow a curve of the form: 3*v*′/2*U*_*c*_ = 0.117 exp(−0.33*r*/*r*_1/2_)^2^ ln(2), whilst the radial velocity fluctuations at *Re* = 135 follow: 3*v*′/2*U*_*c*_ = 0.174 exp(−0.4*r*/*r*_1/2_)^2^ ln(2). The data at x^*^ = 25 shows considerable scatter, indicating that the flow field is still developing into a self-similar structure at this streamwise distance (*i.e.* the velocity data does not collapse to a single curve). Furthermore, data for *Re* = 135, Fig. 4 (a, b; black symbols) does not collapse with data at higher *Re*. This is most likely due to the data at *Re* = 135 being located in the intermediate jet development region (see Table 1), which may not retain a self-similar turbulence structure^9^,^29^. Generally, the data for *Re* = 260 and 400 show good agreement with the helium axisymmetric jet flow data of Amielh *et al.*^24^ (grey solid and dashed lines). Further comparison is made with the helium jet flow data which is free from plasma at *Re* = 400, x^*^ = 70 (green closed circles), in Fig. 4(d). These data show that the radial profiles of the mean and higher order statistics collapse to the plasma data once scaled appropriately.

## Summary

In this letter we report on the characteristics of an inhomogeneous axisymmetric turbulent jet flow generated downstream of a low temperature atmospheric pressure plasma plume. We have shown that the mean turbulent velocity fields of the jet flows under investigation are independent of applied voltage. Indeed, an order-of-magnitude analysis shows that the temperature and electro-hydrodynamic forces generated by the plasma have a negligible effect on the mean velocity fields. These results allow us to posit that the production of turbulence is due to a combination of the small-amplitude electro-hydrodynamic body forces and gas heating generating perturbations in the highly unstable shear layers at the jet exit, which grow as they move downstream, creating Reynolds shear stresses and thus the production of turbulence. Finally, and more broadly, by using a plasma as a unique excitation source, velocity measurements have been conducted in inhomogeneous axisymmetric turbulent jets at Reynolds numbers on the order of 100, which are one order of magnitude lower than any other study available within the literature. Even at such low Reynolds numbers, we have shown that the spatially developing velocity fields are turbulent and self-similar. Furthermore we have shown that the developing self-similar turbulent velocity fields generated by the plasma are similar to the turbulent velocity fields which develop naturally (i.e. without a plasma) with increasing distance from the jet exit. These data provide vital information for the future development of low-temperature atmospheric pressure plasma plume devices and for the validation of associated computational models.

## Methods

**Figure 5:**
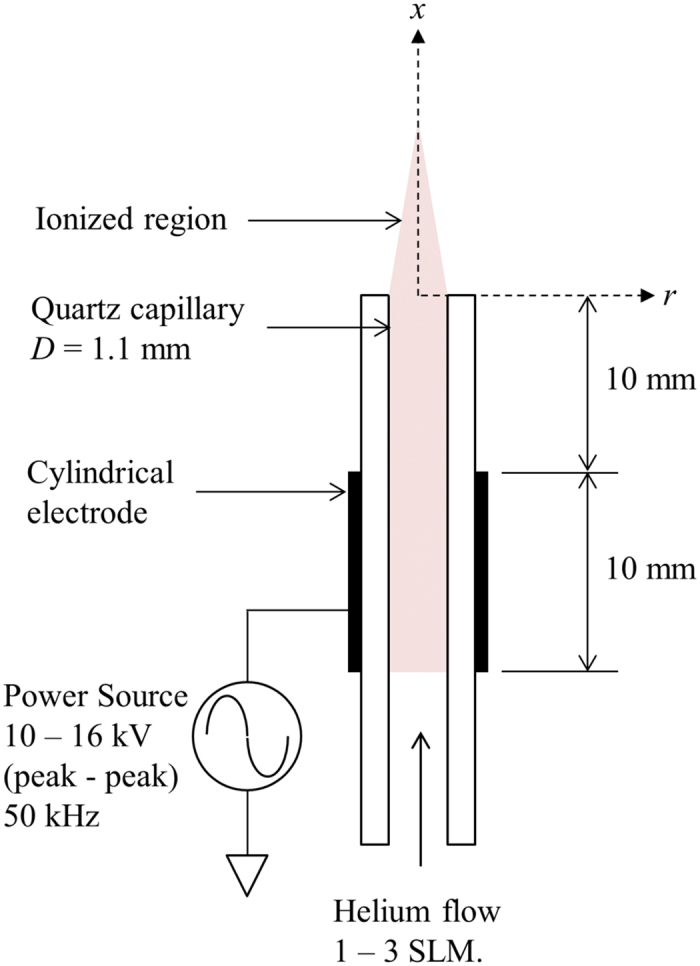
Schematic representation of the experimental set up of the low temperature atmospheric pressure plasma plume device.

The low temperature atmospheric pressure plasma device investigated in this study is shown schematically in Fig. 5. The device consists of a quartz tube with an inner diameter (*D*) of 1.1 mm. The discharge was generated via a 10 mm wide copper electrode, which was wrapped around the outer circumference of the quartz tube and positioned 10 mm away from the jet exit. A custom-built power source applied a sinusoidal voltage waveform to the copper electrode at a voltage of 10–16 kV peak-to-peak at an AC driving frequency of 50 kHz. The applied voltage generated a stable plasma which protruded from the quartz tube within a flow of helium; in this single electrode configuration the electrical circuit is completed via capacitive coupling to ground. An MKS mass flow controller regulated the flow of helium (99.996% purity) at 1, 2 or 3 Standard Litres per Minute (SLM). This provided a range in Reynolds number of *Re* = *U*_*m*_*D*/*ν*_*j*_ = 135–400 (where *U*_*m*_ is the jet exit velocity determined by mass continuity and *ν*_*j*_ is the kinematic viscosity of helium). At a given standard flow rate, the jet exit Reynolds number (*Re*) monotonically decreased with increased applied voltage due to a small increase in mean fluid temperature at the jet exit (between 10–30 degrees Celsius increase above the ambient gas temperature with increasing applied voltage, measured by an Omega FOB100 fibre optic thermometer system). The nominal experimental conditions are shown in Table 1 with (±) range of *Re* as shown.

**Figure 6:**
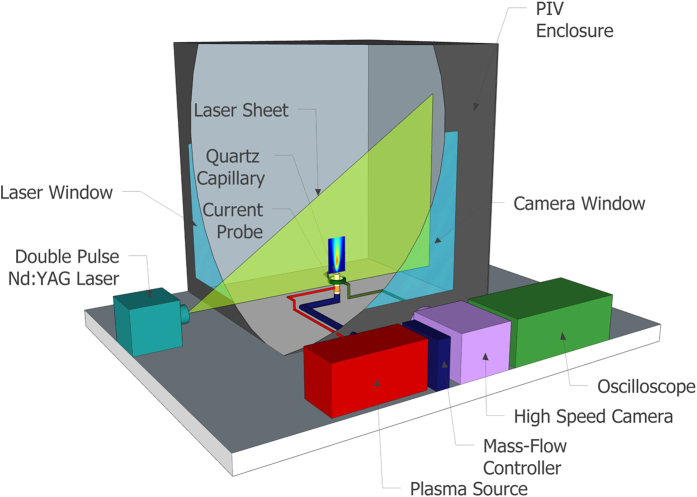
Schematic representation of the experimental set up of the experimental setup.

Global measurements of the velocity field were taken along the centreline of the jet with a time-resolved particle image velocimetry (PIV) system from TSI Incorporated. All measurements were conducted inside a closed chamber to ensure the helium jet flow was not influenced by any external drafts. The chamber had a cross-section of 1000*D* × 1000*D* and a height of 1500*D*. Figure 6 shows a schematic representation of the experimental setup, showing the plasma plume device positioned in the laser sheet within the PIV enclosure. Olive oil with a nominal size of 1 μm was used to seed the air. The Stokes numbers of the seeding particles used throughout this study were <0.1. This ensured that the seeding particles followed the fluid flow closely with tracing errors being <1%^30^. In total, 500 vector maps were acquired at a frequency of 500 Hz for each experiment. The velocity vectors were computed on a square grid with spatial resolution of 0.43*D* using a recursive cross-correlation technique. Finally, the flow field was visualized using Schlieren photography with a Nikon D7000 camera at a shutter speed of 1/80 s; for details of this setup see Ghasemi *et al.*^13^.

## Additional Information

**How to cite this article**: Whalley, R. D. and Walsh, J. L. Turbulent jet flow generated downstream of a low temperature dielectric barrier atmospheric pressure plasma device. *Sci. Rep.* **6**, 31756; doi: 10.1038/srep31756 (2016).

## Figures and Tables

**Table 1:** Nominal experimental conditions.

*Re*	*Fr*	*x*_*I*_/*D*	*f*_0_ (kHz)	*U*_*m*_ (m/s)
135 ± 5	4,300 ± 75	20	27	17.5
260 ± 10	15,450 ± 300	38	51	33.1
400 ± 25	38,800 ± 1,200	60	81	52.6
